# Biomaterial-Driven Integrated Therapy for Renal Cell Carcinoma: Renal Cancer Control and Kidney Injury Repair

**DOI:** 10.34133/research.1188

**Published:** 2026-04-08

**Authors:** Jiaao Song, Tinglin Zhang, Mingmin Li, Wenqiang Liu, Tong Chen, Huifen Qiang, Linhui Wang, Zhenjie Wu, Jie Gao

**Affiliations:** ^1^ Department of Urology, Shanghai Changhai Hospital, Naval Medical University, Shanghai 200433, China.; ^2^ Changhai Clinical Research Unit, Shanghai Changhai Hospital, Naval Medical University, Shanghai 200433, China.; ^3^ Shanghai Key Laboratory of Nautical Medicine and Translation of Drugs and Medical Devices, Shanghai 200433, China.; ^4^ Department of Radiology, Shanghai Changhai Hospital, Naval Medical University, Shanghai 200433, China.

## Abstract

Renal cell carcinoma (RCC), a highly heterogeneous malignancy that arises from the renal tubular epithelium, has exhibited a rising incidence in recent years and has become the predominant urologic malignancy, posing a major threat to human health and life. Although our understanding of RCC has advanced, treatment remains challenging owing to low specificity and sensitivity, particularly with respect to postoperative recurrence and therapy-induced renal injury. Therefore, effective strategies that can improve therapeutic outcomes are urgently needed. In recent years, the expanding investigation of materials for biomedical applications has identified several constructs with promising clinical potential; compared with traditional drugs, these materials offer superior targeting, an improved safety profile, and a broader array of functions and synergistic effects that can effectively overcome RCC recurrence, drug resistance, and metastasis. Moreover, biomaterials can attenuate renal damage and inhibit renal fibrosis, thereby preserving renal function. In this review, we present a comprehensive examination of the current landscape and future directions of biomaterials for RCC and renal injury treatment. We propose an integrated antitumor and kidney protection strategy and establish an analytical framework rooted in the fundamental contradictions of material design, encompassing functional conflicts, stimuli-responsive activation, and metabolic safety. This approach provides novel insights into overcoming critical challenges in the field.

## Introduction

Renal cell carcinoma (RCC), the third most common urogenital malignancy, accounts for 2.2% of all newly diagnosed cancers worldwide [1], and its incidence continues to rise annually [2]. In China, metastatic RCC claims approximately 20,000 lives each year [3]. Although radical nephrectomy can eliminate localized lesions, more than 50% of patients eventually experience recurrence or metastasis [4]. Metastatic RCC remains refractory to conventional chemotherapy and radiotherapy (RT); first-line therapy currently consists of immune checkpoint inhibitors (ICIs) combined with tyrosine kinase inhibitors (TKIs). While TKIs and ICIs have markedly improved outcomes, the majority of patients either harbor primary resistance or acquire resistance after an initial response [5,6]. Moreover, these agents nonspecifically reactivate systemic immunity to achieve antitumor efficacy, resulting in unsatisfactory response rates and increased immune-related adverse events stemming from off-target immune activation [6,7]. Ravi et al. [8] further reported that 95% of metastatic RCC patients discontinued immunotherapy, 72% because of disease progression and 23% because of toxicity.

**Fig. 1.**
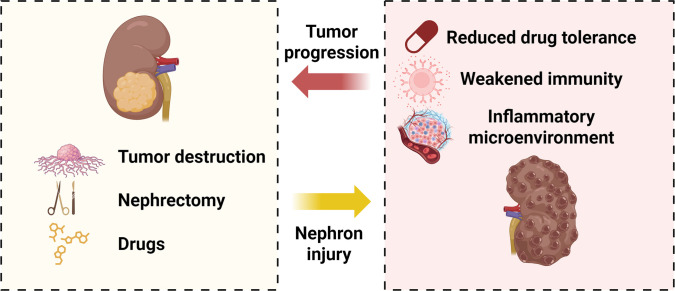
Schematic diagram of the vicious cycle between RCC and renal injury. RCC patients often suffer concurrent renal injury caused by tumor destruction, surgery, and nephrotoxic drugs, which leads to declining renal function. The injured kidney then promotes tumor progression by impairing drug tolerance, immune competence, and the inflammatory tumor microenvironment, creating a vicious cycle (created with BioRender).

During partial nephrectomy for early-stage RCC, renal hilar clamping is frequently needed, imposing ischemia–reperfusion injury that escalates in direct proportion to warm ischemia time [9]. Patients with advanced RCC often present with varying degrees of renal impairment attributable to tumor infiltration, mass effect, therapy-related insults (cytoreductive surgery or systemic agents) and concurrent medical comorbidities. Moreover, renal injury can also affect the progression of RCC. Both acute kidney injury (AKI) and chronic kidney disease (CKD) are epidemiologically linked to RCC development; compared with the general population, RCC patients are more likely to harbor baseline CKD at diagnosis and throughout treatment [10]. The convergent progression of these pathologies can culminate in end-stage renal disease (ESRD), further compromising oncological outcomes [11]. There is a mutually reinforcing relationship between RCC and renal injury, and the 2 can form a vicious cycle, leading to the continuous deterioration of the patient’s health condition (Fig. 1). Several large-scale clinical studies have indicated that in long-term follow-up observations, the remaining unilateral kidneys of kidney donors who underwent unilateral radical nephrectomy were able to maintain normal renal function [12]. In view of this, radical nephrectomy has become the standard surgical method for treating local renal masses. However, the conditions of patients with RCC are significantly different from those of kidney donors. Patients with RCC are usually older and often have risk factors such as diabetes, hypertension, smoking, and obesity, which make their kidney function more vulnerable to damage [13,14]. Some patients even have kidney diseases from the very beginning [15]. Multivariate analysis by Huang et al. [16] indicated that radical nephrectomy remains an independent risk factor for new-onset CKD. In addition, the percentage change in the estimated glomerular filtration rate (eGFR) was significantly correlated with the degree of glomerular sclerosis (*P* = 0.034). For every 10% increase in the degree of glomerular sclerosis, the postoperative eGFR decreased by 9% compared with the preoperative baseline [17]. Meanwhile, there is a bidirectional association between CKD and RCC. Advanced CKD or ESRD, as well as acquired cystic kidney disease (ACKD), are all risk factors for RCC [18]. Therefore, in the treatment of RCC, taking into account the protection of renal function can not only effectively prevent the occurrence of adverse renal outcomes but also significantly improve the quality of life of patients. Current management of renal injury necessitates high intrarenal concentrations of corticosteroids or nonsteroidal anti-inflammatory drugs, yet these agents merely retard—rather than halt—the advancement of renal complications, exhibit limited efficacy, and are constrained by cumulative toxicities that preclude prolonged use [19]. Consequently, the development of an integrated therapeutic strategy that concurrently addresses tumor eradication and renal protection is imperative. However, this dual objective presents 3 fundamental design challenges at the biomaterials level: (a) Functional conflict: the pro-oxidant, pro-apoptotic mechanisms required for antitumor efficacy are inherently at odds with the antioxidant, anti-inflammatory, and reparative functions needed for nephroprotection. (b) Spatiotemporal targeting: these opposing functions must be spatially segregated to the tumor site versus healthy renal tissue, or temporally controlled to avoid interference. (c) Biosafety and clearance: the kidney, as a primary clearance organ, is particularly vulnerable to material accumulation and off-target toxicity, necessitating designs that ensure timely metabolic clearance or biodegradation. This review will analyze current biomaterial systems through the lens of these challenges and propose rational design paradigms for next-generation integrated platforms.

Biomaterials hold considerable promise for the treatment of renal disease, and a diverse array of nanocarriers has already been translated into experimental practice [20], encompassing metallic substrates [21], inorganic nonmetallic matrices, organic macromolecular scaffolds, and composite systems that integrate multiple constituents [22,23]. These constructs can function as drug delivery vehicles for peptides, ribonucleic acids, and small-molecule therapeutics; by enabling precise or sustained release profiles, they refine drug biodistribution and pharmacokinetics, thereby potentiating therapeutic efficacy [24]. Furthermore, surface conjugation of targeting ligands endows the carriers with organotropic homing capacity, markedly curtailing off-target toxicities [25]. Certain materials possess intrinsic therapeutic activity or serve as co-carriers for small-molecule drugs, facilitating combinatorial regimens that amplify treatment outcomes [26]. Additionally, selected platforms act as sonosensitizers or photosensitizers, augmenting the synergistic efficacy of sonodynamic and photodynamic anticancer modalities [27].

**Fig. 2.**
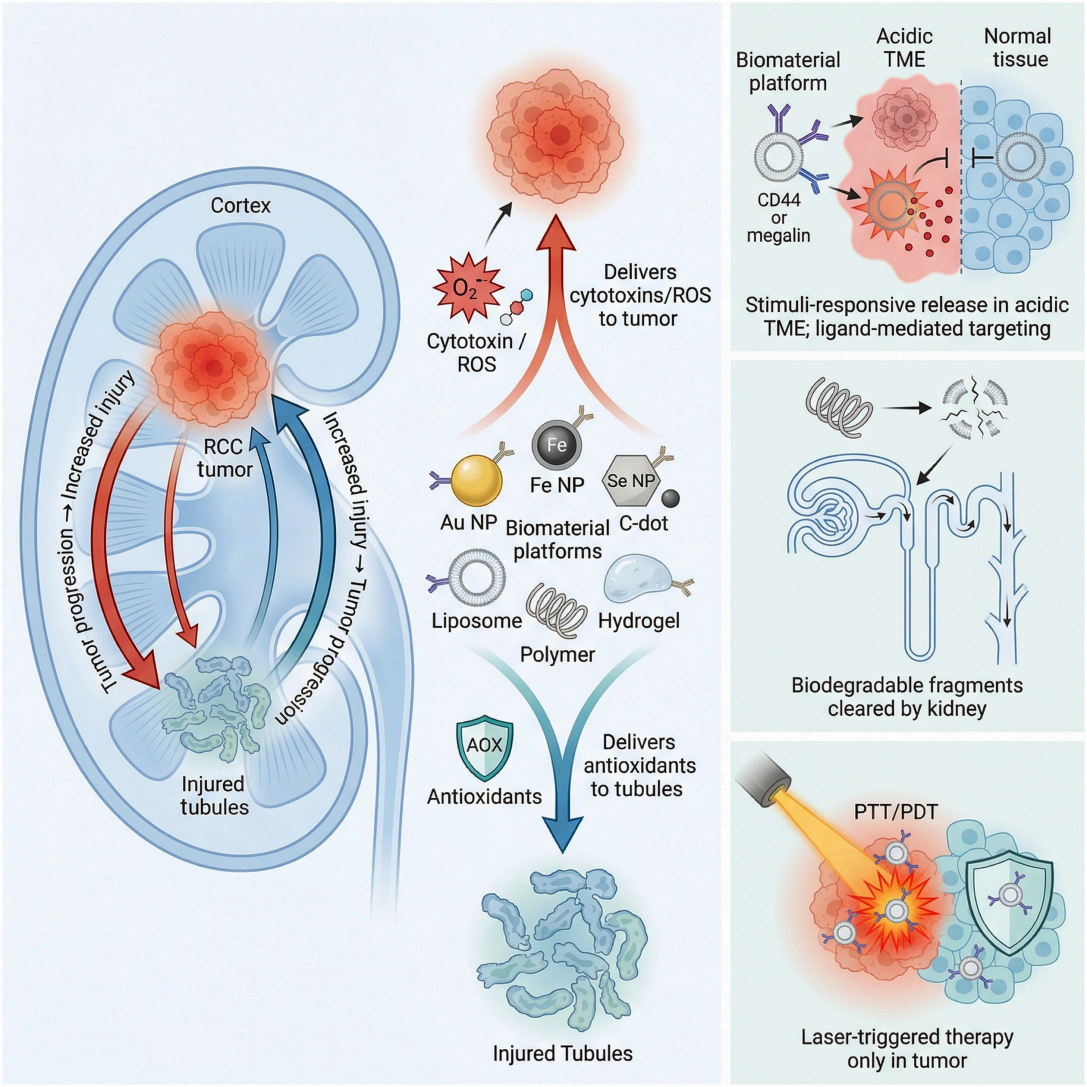
Antitumor and kidney protection integrated strategy. Biomaterials have been widely applied in the treatment of RCC and renal injury. By leveraging a rich library of materials and treatment methods, an integrated “antitumor and kidney protection” strategy can be designed to achieve tumor removal while restoring kidney function. However, several contradictions exist in this integration. The mechanisms involved in antitumor therapy and renal injury repair are distinct or even opposing. Material retention or inherent toxicity may conflict with renal repair objectives. These challenges can be addressed through microenvironment-responsive release, external condition activation (such as ultrasound or light) to enable spatially and temporally distinct functions, and degradable designs that facilitate rapid renal metabolism and eliminate nephrotoxicity. [Assisted by the Research Mechanism Diagram AI Generator (jova.ai). The author has strictly reviewed and modified the artificial intelligence (AI)-generated content, has confirmed its accuracy, and assumes full responsibility for the results.]

The integration of antitumor and kidney protection functions by compounding materials with different functions has promising application prospects in the treatment of RCC (Fig. 2). However, systems simultaneously addressing both RCC and nephroprotection still confront several inherent contradictions. Functional conflicts represent the primary challenge, as the pro-oxidant, pro-apoptotic mechanisms required for tumor eradication are inherently incompatible with the antioxidant, anti-inflammatory, and repair-promoting functions necessary for kidney protection. Balancing these opposing biological activities constitutes the critical determinant for realizing integrated therapeutic strategies. Spatiotemporal targeting challenges constitute the second major hurdle, as these conflicting functions must be segregated temporally or spatially to prevent mutual interference. Enabling materials to execute distinct functions at different sites or time points represents a pivotal technical obstacle in the rational design of such integrated systems. Finally, biosafety challenges must be addressed, given that the kidney serves as the primary route of metabolism and clearance for numerous materials. The inherent metabolic fate, clearance kinetics, and long-term toxicity profiles of materials may conflict with kidney protective objectives, and ensuring the safety profile of the entire material system remains the cornerstone for clinical translation and practical application. Accordingly, this review first summarizes the applications of metallic materials, inorganic nonmetallic materials, and organic materials in the treatment of RCC and kidney injury, examining the contradictions arising from their simultaneous application. Subsequently, based on various promising material platforms, we explore solutions and design strategies to resolve the functional paradoxes inherent to single-material systems. Finally, we emphasize the paramount importance of biosafety and illuminate future development trajectories for biomaterials integrating antitumor efficacy with repair-promoting functionalities.

## Metal Materials for Integrated Therapy

Metal ions play a crucial role in human physiological processes. They are indispensable in key processes, such as maintaining cellular homeostasis, metabolic regulation, material synthesis, signal transmission, and energy conversion [28–30]. However, abnormal distribution or accumulation of metal ions not only leads to damage to cell structure and function but also may trigger a series of biochemical reactions, ultimately inducing cell death [31]. It is worth noting that there is a certain correlation between the occurrence and development of RCC and the changes in metal ion levels. Pirincci et al. [32] found by comparing the serum trace elements of patients with RCC and healthy controls that the levels of cadmium (Cd) and plumbum (Pb) in the serum of patients with RCC were significantly increased, while the levels of zinc (Zn), iron (Fe), and manganese (Mn) were relatively low. Therefore, by stimulating exogenous metal ions or interfering with the functions of endogenous metal ions, the goal of efficiently inhibiting tumor growth can be achieved, and this strategy will not cause drug resistance problems [33]. Meanwhile, metallic materials are widely used in biological imaging and drug delivery due to their unique optical, magnetic, and photothermal properties [34]. At present, new tumor treatment methods, including RT sensitization, photodynamic therapy (PDT), photothermal therapy (PTT), sonodynamic therapy (SDT), and chemodynamic therapy (CDT), all rely on the participation of metal-based nanomaterials [34–37]. The inherent antioxidant capacity of the metal itself can also play a role in protecting against kidney injury [38].

### Gold

Gold (Au) nanoparticles (NPs) have attracted much attention in the field of antitumor biomaterials due to their outstanding biocompatibility, simple preparation process, excellent stability, low toxicity, multivalence, and adjustable size characteristics [39]. These NPs can exert antitumor effects through multiple mechanisms, including efficient drug delivery, activation of immune responses, enhancement of RT sensitivity, and blocking the interaction between tumor cells and vascular endothelial cells, thereby effectively combating the growth and spread of tumors [40]. The search results of Xu et al. [41] indicate that Au is one of the most commonly used antitumor metal nanomaterials in recent years. Callaghan et al. [42] used Au nanorods loaded with TKI to treat RCC. Au also served as a catalyst for photothermal ablation to generate local thermal energy for a combined therapeutic effect, significantly reducing the viability of the RCC cell line 786O cells. After verifying its effectiveness in vitro, they then conducted verification in nude mice. The results showed that after the material was irradiated by a laser in vivo, the cell killing rate reached 100% [43]. Kannadorai et al. [44] utilized the dual capabilities of Au as a photothermal agent and an autofluorescence enhancer to track cell death while treating RCC. Liu et al. [45] synthesized Au nanoparticles (AuNPs) using *Curcuma wenyujin*, which can increase the expression of apoptotic proteins in RCC cell lines, promote tumor apoptosis, and have a good antitumor effect.

AuNPs themselves possess certain anti-inflammatory and antioxidant functions and can be used to treat kidney damage. Reshi et al. [38] found that AuNPs could reverse liver and kidney damage caused by acetaminophen in rats and restore normal values to indicators such as aspartate aminotransferase, alanine aminotransferase, bilirubin, and creatinine. AuNPs also have a protective effect on renal tubulointerstitial injury and can be used to treat antiproteinuria [46]. Al-Tantawy et al. [47] discovered that AuNPs can exert renoprotective effects by inhibiting the expression of miR-192 and miR-21, thereby suppressing downstream pathways of fibrosis, apoptosis, autophagy, and oxidative stress. AuNPs can also be used to carry drugs for treating kidney injury, synergistically enhancing the therapeutic effect on kidney injury. Zhang et al. [48] discovered that developing ultrasmall Au nanoclusters (1 to 2 nm) loaded with N-acetylcysteine (NAC) for the treatment of AKI can effectively address the shortcomings of NAC, such as low bioavailability, low renal accumulation, short renal retention time, and high dose-induced toxicity, protecting the kidneys from oxidative damage and inflammation and promoting the recovery of renal function. Furthermore, AuNPs can be combined with other metals to construct more complete drugs and exert better anti-damage effects. Ma et al. [49] introduced Cu single-atom active sites to atomic-precision Au22 clusters. The Au21Cu1 clusters they constructed can increase the antioxidant activity of Au by 18 times. The catalase-like activity increased by 90 times, and the superoxide dismutase (SOD)-like activity increased by 3 times. It can effectively inhibit oxidative stress and inflammation in the kidneys and can be visualized in 3-dimensional space through near-infrared II (NIR-II) light sheet microscopy.

The excellent NIR response of AuNPs makes them commonly used as photosensitizers in PDT and PTT therapies. Their outstanding surface properties enable precise surface functionalization. Moreover, their chemical inertness ensures good biological safety. However, there are still issues in integrated therapy. Their excellent chemical inertness makes them difficult to degrade and excrete from the body, resulting in a size contradiction. In antitumor treatment, AuNPs with a diameter of 50 nm or above have good photothermal properties and drug loading capacity, but are difficult to excrete from the body, causing accumulation in the liver and spleen. In the treatment of kidney injury, AuNPs with a diameter of 6 nm or less can be quickly excreted from the body through the kidneys, showing good biological safety, but their photothermal conversion efficiency and drug loading capacity are affected. By modifying with degradable materials, developing degradable AuNPs responsive to the tumor microenvironment, and making their size suitable for excretion through the kidneys after degradation can solve the contradiction of AuNPs in integrated treatment.

### Iron

Fe, as the most abundant transition metal in the human body, plays a crucial role in basic physiological processes such as DNA synthesis and mitochondrial respiration and is closely related to the occurrence and development of RCC [50,51]. Studies have shown that compared with healthy individuals, the expression levels of some Fe-regulated genes in patients with RCC are significantly elevated, and this abnormal expression is closely related to a poor prognosis [52]. Clinical evidence and experimental models also indicate that Fe overload in the kidneys is associated with an increased incidence and metastasis rate of RCC [53,54]. Schnetz et al. [55] found that macrophages can lead to an increase in Fe content both within and outside tumor cells, thereby promoting the proliferation and migration of tumor cells. The role of Fe nanoparticles (FeNPs) in kidney protection mainly stems from their nano-enzyme design and the Fe element’s own ability to alleviate kidney injury [56].

Magnetic NPs (MNPs), as a class of submicroscopic solid particles, have a core composed of Fe oxide (Fe_2_O_3_) and are usually encapsulated in polymers, silica, and organic materials, aiming to enhance their colloidal stability and encapsulation efficiency [57]. MNPs, with their unique biocompatibility, chemical stability, and ease of functional modification, significantly outperform other antitumor drug nanocarriers [58]. Takke and Shende [59] innovatively used Fe_2_O_3_ NPs as magnetic cores and encapsulated them in polylactic acid-polyglycolic acid (PLGA) for the delivery of silibinin to treat RCC. This system demonstrated a continuous release characteristic of up to 15 d in vitro and showed a significant killing effect against RCC cells, being more effective than silibinin alone. Grillone et al. [60] further explored the potential application of the magnetic properties of Fe_2_O_3_ in tumor treatment. They achieved precise drug delivery to the lesion site by co-encapsulating Fe_2_O_3_ and sorafenib in cetylpalmitate with the assistance of a remote magnetic field. This strategy not only significantly enhances the therapeutic effect of the drug but also effectively reduces its side effects. In addition, Fe_2_O_3_ also has the ability to induce endoplasmic reticulum stress (ERS). This ability can disrupt the redox balance within the endoplasmic reticulum lumen, disrupt protein folding, and increase ERS. Therefore, it is regarded as a potential strategy to promote ERS in tumor sites [61]. Cai et al. [61] synthesized an Fe_2_O_3_ NP loaded with the unfolded protein response (UPR) modulator PR-619 to enhance ERS and coupled it with the tumor-homing peptide tLyP-1 to form the tLyP-1/PR-619/Fe_3_O_4_@PCM (tPF@PCM) therapeutic diagnostic platform. This platform can induce irreversible ERS and activate the “end-stage” UPR, thereby leading to apoptosis of cancer cells, and has shown satisfactory photothermal enhanced tumor suppressor effects both in vitro and in vivo.

In RCC, due to the high expression of hypoxia-inducible factor-1α (HIF-1α) and HIF-2α, the level of polyunsaturated fatty acids (PUFAs) abnormally increases, resulting in a significantly higher PUFA level in RCC than in normal renal tissues [62]. PUFAs are more prone to oxidation to form lipid peroxides (L-OOH), thereby making ferroptosis more likely to occur. Based on this feature, Ni et al. [63] synthesized an Fe-based metal organic framework NP loaded with a ferroptosis exciter (RSL3). This particle can target RCC cells through a pH response, release the loaded Fe and RSL3, induce Fe overload, and inhibit glutathione peroxidase 4 (GPX4), thereby triggering a cascade ferroptosis reaction and killing tumor cells. These studies have shown that Fe plays multiple roles in tumor treatment, including serving as a drug carrier, inducing ERS, and promoting ferroptosis, providing new strategies and targets for tumor treatment.

The systemic Fe metabolism of patients with CKD is disrupted. Intracellular Fe deficiency in renal macrophages leads to oxidative stress in macrophages, causing proinflammatory polarization and increasing the production of proinflammatory cytokines. Fe therapy, such as ferric dextran, can alleviate renal fibrosis and has a certain protective effect on the kidneys. Therefore, targeting intracellular Fe deficiency in renal macrophages in CKD can serve as a therapeutic opportunity to alleviate disease progression [64]. At present, there are clinical trials exploring the effects of intravenous Fe preparations on oxidative stress and renal injury markers in CKD [65]. Duan et al. [56] designed ultrasmall Fe_3_O_4_ NPs for delivering nicotinamide mononucleotide (NMN), a precursor of nicotinamide adenine dinucleotide (NAD), which can enhance mitochondrial function and inhibit inflammation. Fe_3_O_4_ may treat AKI by promoting erythropoiesis through Fe supplementation. Xu et al. [66] conjugated Fe sulfide with glutathione (GSH) to prepare Mackinawite nanozyme, which possesses multiple enzyme-like properties and hydrogen polysulfide release characteristics. The synergistic interaction between the 2 demonstrates broad-spectrum reactive oxygen species (ROS) scavenging ability and can show excellent protective effects against ROS-induced renal injury at extremely low doses.

In the field of biomedical materials, FeNPs can be classified into zero-valent Fe, ferroferric oxide, ferrous oxide, and Fe-based metal–organic frameworks (Fe-MOFs) based on their valence state and crystal structure. Since Fe is a normal trace element in the human body and has corresponding metabolic mechanisms, the safety of FeNPs is much better than that of precious metals. The core value of its application in antitumor lies in Fe death caused by lipid peroxidation and the chemical kinetic property that can trigger the Fenton reaction. However, this property also contradicts the need for antioxidant and Fe homeostasis protection in the kidneys. Designing stimuli-responsive release systems based on the differential pH, GSH levels, and other biochemical gradients between the tumor microenvironment and normal tissues offers an effective solution to this challenge. At the same time, Fe₃O₄ is a compound with mixed valence states, having both oxidizing and reducing properties, which can provide ideas for integrated treatment.

### Copper

As a double-edged trace metal, copper (Cu) is indispensable for normal physiology yet intrinsically toxic through its capacity for redox cycling. Especially during the growth and metastasis of tumors, the demand for Cu increases significantly. Studies have shown that compared with normal tissues, the concentrations of elements such as calcium (Ca), Cd, potassium (K), magnesium (Mg), Mn, sodium (Na), Pb, sulfur (S), and strontium (Sr) in RCC tissues are significantly increased, while the concentration of Cu is particularly prominent in young patients [67]. This phenomenon suggests that Cu metabolism may become a potential target for the treatment of RCC. In terms of kidney protection, similar to FeNPs, it also achieves antioxidant effects and alleviates kidney injury through the SOD-like activity of small-sized NPs.

**Fig. 3.**
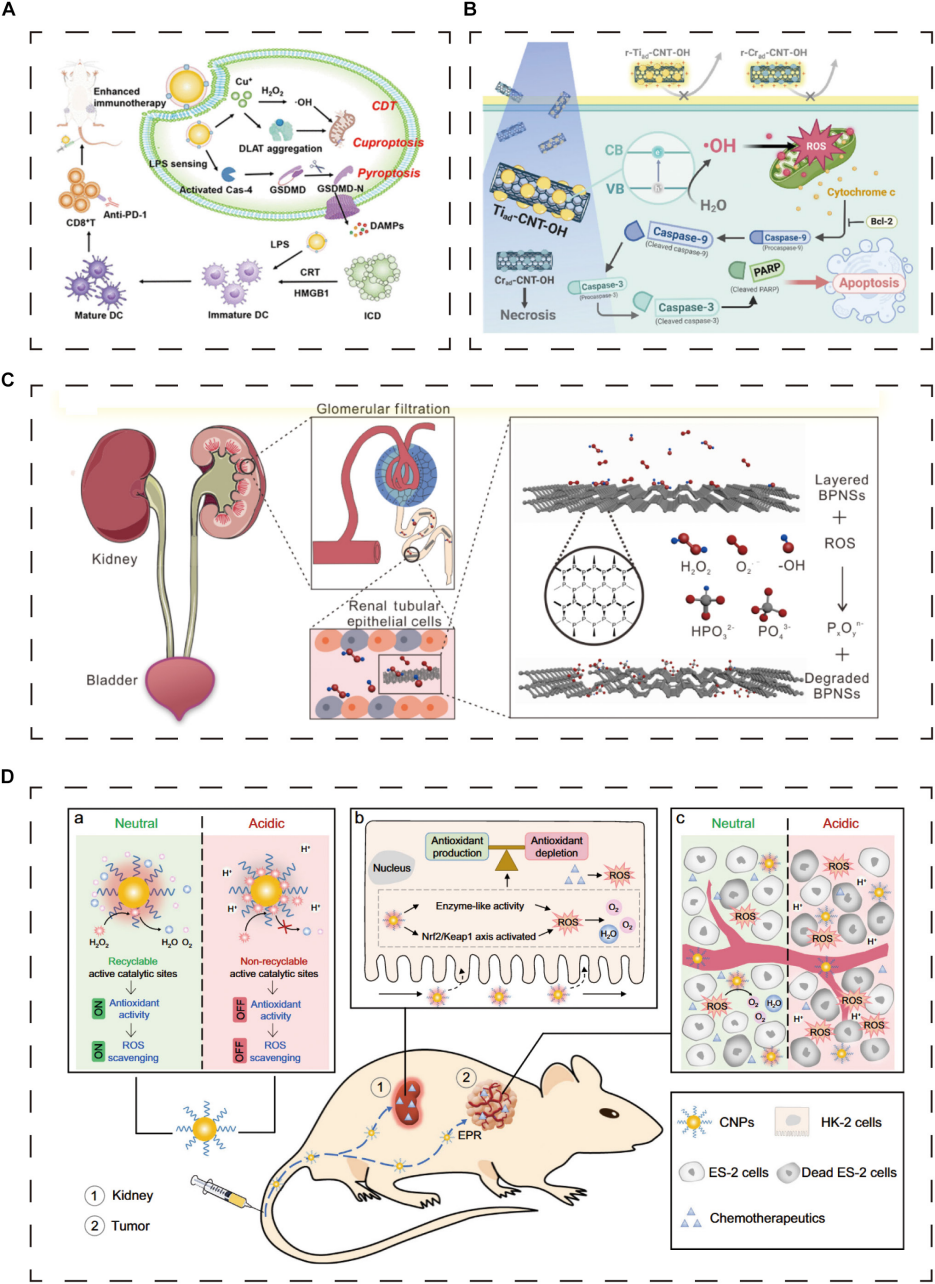
Different types of materials used to treat RCC and kidney injury. (A) Metal materials are used to treat RCC. (B) Inorganic nonmetallic materials are used to treat RCC. (C) Inorganic nonmetallic materials are used to treat kidney injury. (D) Integrated therapy of tumor and kidney injury. (A) Reproduced with permission from [69]. © Wu MT (2025). (B) Reproduced with permission from [87]. © Park HB (2025). (C) Reproduced with permission from [118]. © Hou JJ (2020). (D) Reproduced with permission from [185]. © Weng QJ (2021).

Yang et al. [68] ingeniously took advantage of the elevated Cu content in RCC tissues and designed cuprous oxide NPs (CONPs). This kind of NP can interfere with the Cu transport process by regulating Cu chaperone proteins in RCC cells, thereby disrupting the Cu metabolism of tumors. The mechanism of action of CONP is not limited to regulating Cu metabolism. It can also induce the ERS response by promoting the accumulation of intracellular Ca ions and ROS in both in vitro and in vivo experiments. More importantly, CONP can initiate ER and mitochondrial-dependent apoptotic pathways by activating apoptosis-related proteins such as caspase-3, caspase-9, and caspase-12. This discovery provides a new perspective for the application of Cu in tumor treatment. Specifically, the application of CONPs not only reveals the crucial role of Cu in tumor growth but also provides a theoretical basis and practical guidance for the development of Cu-based tumor treatment strategies. Chen and colleagues [69] reported a Cu₂O biomimetic NP that induces immunogenic cell death through the synergistic promotion of cuproptosis and inflammatory cell death, thereby driving dendritic cell maturation and cytotoxic T lymphocyte infiltration, amplifying the efficacy of ICIs and suppressing RCC progression (Fig. 3A).

The ultrasmall Cu-based NPs (CuNPs) synthesized by Liu et al. [70] possess the characteristics of catalase, SOD, and glutathione peroxidase (GPx) mimics. They have a protective effect on ROS-mediated cell damage at extremely low doses and can significantly improve the therapeutic effect of AKI. It can also be quickly excreted through the kidneys and has good biocompatibility and extremely low toxicity. The Cu–niacin complex synthesized by Medhat Hegazy et al. [71] can restore renal dysfunction caused by glycerol and resist the effects of oxidative stress and fibrosis on the kidneys.

Similar to FeNPs, CuNPs can also exert tumor-killing effects through the Fenton-like effect and induce CDT. At the same time, Cu can also induce a special form of cell death called Cu death. Moreover, the designs for alleviating kidney injury in both cases are similar. They both utilize the SOD-like activity of small-sized NPs and facilitate rapid excretion through the kidneys, thereby reducing oxidative stress and minimizing toxic side effects. Therefore, the therapeutic trade-offs inherent in both scenarios share significant similarities, allowing for cross-fertilization of design principles when developing integrated therapy strategies.

### Zinc

Zn is the second most abundant trace element in the human body after Fe and is also the most abundant element in cells [72]. Zn can affect tumor angiogenesis by influencing the level of HIF-1α and the synthesis of various growth factors and inhibit tumor growth and metastasis [73]. HIF-1α is a key molecule in the occurrence and development of RCC and has become a target for the clinical treatment of RCC. Therefore, Zn has great potential in the treatment of RCC [74]. Zinc oxide NPs (ZnO-NPs) have been approved by the Food and Drug Administration (FDA) for cancer treatment and have good biocompatibility [75]. El-Sonbaty et al. [76] synthesized ZnNPs using edible fungi as raw materials to treat RCC, which can effectively reduce the level of caspase-3 and the concentration of carcinoembryonic antigen and has a good antitumor effect.

Zn not only plays an antitumor role in RCC but also has a powerful protective effect on the kidneys. ZnO-NPs can protect against kidney damage caused by various factors through multiple effects, such as antiapoptotic, anti-inflammatory, and antioxidative effects. Barakat et al. [75] treated cisplatin-induced renal injury with ZnO-NPs. Compared with the control group, ZnO-NPs could significantly down-regulate oxidative stress and inflammatory markers and reduce the proportion of apoptosis and necrosis, demonstrating the renal protective effect of the cisplatin drug. The research by Awadalla et al. [77] also indicates that ZnO-NPs may enhance cell proliferation (Ki67) by inhibiting oxidative stress and up-regulating antioxidant genes (Nrf2, HO-1, and HIF-1α). Down-regulation of inflammatory cytokines [tumor necrosis factor-α (TNF-α)] and apoptotic genes (caspase-3 and Bax) protects against renal injury caused by ischemia–reperfusion. Embaby et al. [78] found that ZnO-NPs can mediate kidney protection by activating Nrf2 and preventing oxidative stress and inflammation induced by nuclear factor κB (NF-κB) activation. Aioub et al. [79] explored the protective effect of ZnO-NPs on abamectin-mediated renal injury. In vivo experiments in rats demonstrated that ZnO-NPs could restore the reduced activity of antioxidant enzymes caused by abamectin and inhibit the expression of cyclooxygenase-2 (COX-2). Saadat et al. [80] explored the protective effect of ZnO-NPs on ionizing radiation (IR) renal injury. The results showed that it could improve the renal function of irradiated mice by reducing oxidative stress factors and renal tissue damage.

The design concept of ZnNPs for antitumor and kidney protection is similar to that of the aforementioned metal elements. However, its chemical properties are more active, requiring stricter process control to regulate its degradation rate. Moreover, its application in RCC and kidney protection is relatively limited. However, it also has unique mechanisms such as zinc finger protein regulation and anti-angiogenesis, which provide different perspectives for the design of integrated treatment.

### The contradictions and balance strategies of metal materials in integrated therapy

Metal NPs are one of the candidate substances for the next generation of antitumor treatment due to their advantages, such as low cost, easy synthesis, and flexible functional regulation. It can have a certain therapeutic effect on RCC by itself and can also be used as a drug carrier to carry drugs for combined treatment, further enhancing the therapeutic effect and reducing side effects. By mediating the effects of PDT, PTT, CDT, etc., it kills tumor cells. It reduces oxidative stress and inflammation to decrease cell apoptosis and alleviate kidney injury (Table 1). However, these 2 functions are in conflict. Therefore, a metal that can simultaneously provide kidney protection and antitumor effects can be used, such as Mn. Mn-based nanomaterials can reshape the immunosuppressive tumor microenvironment (TME) by alleviating tumor hypoxia, promoting macrophage polarization, and eliminating immunosuppressive metabolites [81]. Mn can be involved in the treatment of tumors in various ways, such as serving as a biocompatible nanocarrier, an immune adjuvant, and an activator of the cGAS-STING pathway. At the same time, the stimulus-responsive design can also be utilized to separate the 2 scenarios. By precisely controlling the release of both endogenous triggers (such as pH/ROS/GSH/enzymes) and exogenous triggers (such as light/magnetism/ultrasound), the contradiction between antitumor and kidney protection can be resolved. For instance, the drug BP@CeO_2_-polyethylene glycol (BP@CeO2-PEG) designed by Gao et al. [82], which has pH responsiveness, can exert different functions according to the different pH environments of tumor and normal tissues, thereby achieving the integration of antitumor and organ protection. Meanwhile, there is still a lack of certain standards for its synthesis and preparation. Under the premise of clearly controlling the preparation technology, repeatability, stability, dosage, accumulation levels of target and nontarget sites, and the most critical toxicological hazards, metal NPs will become one of the important means for the clinical treatment of RCC.

**Table 1.** Metal biomaterials for RCC therapy and renal injury repair

Core material	Antitumor function			Nephroprotective function		
	Platform	Antitumor function	Key refs.	Platform	Nephroprotective function	Key refs.
Gold	Gold nanorods	Carrier, photothermal agent	[42,44]	Gold nanoparticles, gold-copper nanoclusters	Carrier, enhance antioxidant activity	[47–49]
Iron	Iron oxide, MOF	Drug delivery, ferroptosis trigger	[60,61,63]	Fe^3^O^4^ nanoparticles	Drug delivery, iron supplementation	[56]
Copper	Cuprous oxide nanoparticles, biomimetic nanozyme	Drug deliver, copper-metabolism disruptor, cuproptosis	[68,69]	Ultrasmall copper-based nanoparticles	Mimics catalase, SOD, and GPx	[70]
Zinc	Zinc oxide nanoparticles	Restores GSH, GPx, SOD activity	[76]	Zinc oxide nanoparticles	Attenuates oxidative stress and inflammation, decreases apoptosis	[75]

GSH, glutathione; GPx, glutathione peroxidase; SOD, superoxide dismutase

Metal-based nanomaterials offer a versatile toolkit for integrated therapy, leveraging their diagnostic capabilities, catalytic activities (e.g., Fenton reactions), and ability to induce unique cell death pathways (e.g., ferroptosis and cuproptosis). Their inherent roles in physiology also provide a rationale for renal protection (e.g., Fe supplementation and antioxidant nanozymes). However, their clinical translation is hindered by persistent concerns regarding long-term biodistribution, potential ion leakage, and nonspecific oxidative damage to healthy tissues, especially the kidneys. The functional conflict between their pro-oxidant (antitumor) and antioxidant (nephroprotective) roles remains the central paradox. Future designs must prioritize stimuli-responsive activity and biodegradable coordination architectures (e.g., MOFs) to confine toxic effects spatially and temporally. Furthermore, standardized synthesis, rigorous pharmacokinetic/toxicokinetic studies, and reproducibility are non-negotiable prerequisites before clinical consideration. Among metals, essential elements like Fe and Mn, with endogenous metabolic pathways, may present a safer starting point for integrated system development.

## Inorganic Nonmetallic Materials for Integrated Therapy

Due to their chemical stability, inorganic nonmetallic nanomaterials primarily serve as drug delivery vehicles or exert auxiliary functions through unique physical properties, such as photosensitizers, contrast agents, or RT sensitizers. This inherent stability circumvents the intrinsic cytotoxicity characteristic of metallic NPs, rendering them suboptimal for direct tumoricidal applications but significantly enhancing biocompatibility. Consequently, they can be employed without restriction in kidney injury therapy, unencumbered by material-specific toxicities, forming a complementary strategy to metallic nanomaterials.

### Carbon

Activated carbon nanomaterials (CNMs), with their unique 6-membered carbon ring structure, can form π–π interactions and hydrophobic interactions with drug molecules. This characteristic shows broad application prospects in the biomedical field, especially in drug delivery systems [83]. The family of activated CNMs includes various forms, such as carbon nanotubes (CNTs), graphene, and graphene oxide (GO) nanosheets [84]. Among them, GO materials have shown great potential in the field of tumor treatment due to their excellent NIR absorption characteristics, high dispersibility, good colloid stability, tunable surface functionalization, and outstanding biocompatibility [85]. Jiang et al. [84] ingeniously combined reduced GO (rGO) with gallic acid and used radiofrequency radiation (RF) to treat RCC. They found that this combined strategy exhibited significant apoptotic and toxic effects on A489 RCC cells, and its efficacy was even superior to that of RF treatment alone. CNTs are renowned for their unique physicochemical properties and tubular structure. Their large surface area and ease of processing and modification enable CNTs to be loaded with drugs through various functionalization methods [86]. Park et al. [87] reported that diverse modified OH-functionalized CNTs (CNT-OH) can enhance photocatalytic activity, elevate ROS levels, and promote apoptosis in RCC cells, offering favorable targeting and safety (Fig. 3B). Fisher et al. [88] further demonstrated the potential of multiwalled CNTs (MWCNTs) in the treatment of RCC. The MWCNTs they synthesized demonstrated superior performance in enhancing laser thermal deposition compared to single-walled CNTs (SWCNTs), which not only effectively killed Renca cells but also reduced the expression of heat shock proteins (HSPs), thereby enhancing the therapeutic effect of RCC.

In the field of renal injury treatment, carbon quantum dots (CDSs), as a novel type of nanocarrier, have demonstrated significant roles in targeted drug delivery to the kidneys, elimination of oxidative stress, and inhibition of apoptosis. Zhu et al. [89] loaded deferoxamine with PEG-modified CDSs, where CDSs could enhance the kidney-targeting ability of deferoxamine and exert antioxidant effects. This nanosystem could inhibit ferroptosis and apoptosis by suppressing labile Fe species and eliminating ROS, thereby alleviating AKI caused by cisplatin. The m-phenylenediamine-based CD designed by Gao et al. [90] can preferentially accumulate in the injured kidneys of AKI mice induced by ischemia–reperfusion (IR) and has the property of scavenging multiple toxic free radicals, which can effectively protect cells under various oxidative stresses. GO has demonstrated unique application potential in the field of renal injury treatment, especially in therapeutic strategies for cisplatin-induced AKI. Foroutan et al. [91] jointly injected GO and conditioned medium from mesenchymal stem cells (MSCs) for the treatment of cisplatin-induced AKI. GO can enhance the improvement of renal function and renal morphology by adhering to cytokines in the conditioned medium of MSCs. Moreover, when GO is applied alone, it can also accelerate the improvement of cisplatin-induced AKI. Karimzadeh et al. [92] directly injected GO and MSCs into AKI rats, which also demonstrated significant improvements in renal function and renal morphology. Graphene has demonstrated multiple biological effects in the treatment of kidney injury. It not only enhances the therapeutic effect through the combined application of MSCs but also may directly act on damaged kidney tissues to promote inflammation regulation, immune regulation, angiogenesis, and antioxidant stress, thereby playing a significant role in promoting kidney repair and restoring function. This discovery provides a new research direction and potential therapeutic strategies for the application of graphene in the treatment of kidney diseases. CNTs and their derivatives have shown significant potential in the field of renal injury treatment, especially in intervention strategies targeting the complex pathological process of renal fibrosis. Wang et al. [93] successfully inhibited the process of renal interstitial fibrosis by delivering single-stranded DNA with miRNA-382 inhibitor function to the kidneys using SWCNTs as delivery vectors. This research not only reveals the multifunctionality of graphene and its derivatives in biomedical applications but also provides new ideas for precisely regulating the microenvironment of the kidneys using nanotechnology. Furthermore, Alidori et al. [94] developed an ammonia-functionalized CNT (fCNT), which efficiently transports Trp53 and Mep1b-targeted siRNA to renal proximal tubular cells. It significantly reduced the occurrence of renal fibrosis and verified its good biodistribution characteristics in nonhuman primate models, indicating its great potential in clinical applications. This research not only verified the efficient transport capacity of ammonia-functionalized CNTs in the treatment of kidney diseases but also effectively reduced the expression of fibrosis-related genes by precisely regulating the expression of key genes, providing strong evidence for the application of graphene and its derivatives in the treatment of kidney diseases.

CNMs share similar applications with AuNPs in integrated therapy. Possessing excellent chemical stability and biocompatibility, CNMs primarily serve as drug delivery vehicles, while their unique physicochemical properties confer superior drug loading capacities. Additionally, their favorable optical characteristics enable them to function as photosensitizers, inducing PDT and PTT for tumor eradication. However, the problems faced by both are also quite similar. The stable chemical stability can easily lead to accumulation in the body. Furthermore, the specific sheet-like or tubular morphologies of CNMs introduce distinct toxicological risks. MWCNTs with high aspect ratios (>20:1) are classified as group 2B possible carcinogens by the International Agency for Research on Cancer (IARC) [95]. Consequently, rational design strategies should incorporate size-switchable features that enable in vivo degradation from macromolecular assemblies into small molecular fragments, thereby facilitating renal clearance and mitigating size-dependent toxicities.

### Selenium

Se is a micronutrient in the human diet that participates in cell metabolism, the regulation of DNA and RNA, and protein synthesis, and it is the active site of several enzymes in the antioxidant network [96]. Studies have shown that the content of Se in plasma is negatively correlated with the occurrence of RCC [97], and it may have a protective effect on the development of RCC by inhibiting oxidative DNA damage and tumor growth [98]. Se has also been explored in clinical practice as a drug for inhibiting the growth of RCC and improving drug resistance [99]. Se nanoparticles (SeNPs) have excellent bioavailability and low toxicity, making them ideal carriers for antitumor drugs [100,101]. Jiang et al. [100] synthesized SeNPs modified by *Oudemansiella raphanipies* polysaccharide (ORPS), which can effectively inhibit the proliferation of 4 cancer cell lines, among which the inhibition of RCC cell lines is the most obvious. Apoptosis is induced through ROS imbalance and mitochondria-mediated pathways and ultimately leads to oxidative stress damage in cells.

Se is a cofactor for multiple enzymes (selenoproteins) and is deeply involved in thyroid hormone metabolism, enzymatic antioxidant defense, and the regulation of the immune system [102]. There is a close and clear connection between the kidneys and Se metabolism. Many Se proteins are synthesized in the kidneys, such as type 1 5′-deiodinase (D1) and renal and plasma GPx [103]. Severe Se deficiency can lead to a reduction in D1 and GPx proteins and their activities in a tissue-specific manner, while Se supplementation can increase them [104]. Conditions such as AKI, CKD, and ESRD are usually closely related to lower serum and tissue Se levels [105]. A large number of research results have shown that Se has a powerful protective effect on kidney injury [106–108]. Research by Chen et al. [109] revealed that in areas with higher levels of Cd and Se in the soil, the population did not show more severe kidney damage. This might be attributed to the fact that Se can combine with free Cd, thereby activating the antioxidant enzyme system and exerting its protective effect. SeNPs exhibit higher biocompatibility than inorganic and organic forms of Se, thus making them an ideal alternative to nutritional supplements [110].

AlBasher et al. [111] treated glycerol-induced AKI with SeNPs and found that SeNPs could effectively prevent the development process of AKI by means of antioxidant, anti-inflammatory, and anti-apoptotic activities and significantly alleviate the biochemical, molecular, and histological changes caused by glycerol. The SeNPs synthesized by Mehanna et al. [112] can significantly reduce the levels of malondialdehyde (MDA), inducible nitric oxide synthase (iNOS), nitric oxide (NO), TNF-α, and kidney injury molecule-1 (KIM-1). It also significantly enhances the activity of antioxidant enzymes and mitochondrial enzyme complexes in the kidneys. Liu et al. [113] developed spore oil-functionalized nano-selenium (GLSO@SeNPs), which can inhibit mitochondrial apoptosis by maintaining oxidative homeostasis and regulating related signaling pathways [mitogen-activated protein kinase (MAPK), caspase, and AKT signaling pathways]. Inflammation is inhibited simultaneously by reducing the proportions of CD3^+^CD4^+^ T cells, CD3^+^CD8^+^ T cells, and M1-type macrophages and increasing the proportion of anti-inflammatory regulatory T cells. Rosenkrans et al. [114] synthesized Se-doped carbon quantum dots (SeCQDs), and the results showed that SeCQDs had broad-spectrum antioxidant properties and significant renal accumulation in AKI mice. Its therapeutic effect exceeded that of amifostine, demonstrating great therapeutic potential. Se plays a crucial role in the treatment of kidney injury. Through multiple mechanisms, including antioxidation, anti-inflammation, anti-apoptosis, and regulation of the immune system and signaling pathways, it provides powerful protection for the kidneys and has great potential in the treatment of kidney diseases.

As an essential trace element for humans, SeNPs occupy the interface between nutraceuticals and nanomedicine, featuring physiological metabolic pathways and serving as core cofactors for various antioxidant enzymes. They have found extensive applications in alleviating kidney injury and demonstrate a negative correlation with RCC progression, making them suitable for integration into unified therapeutic systems. Importantly, they complement other materials without increasing overall systemic toxicity. Furthermore, the incorporation of responsive diselenide bonds endows the system with tumor microenvironment-responsive controlled release characteristics, enhancing targeting specificity. The degradation of these diselenide bonds reduces material dimensions, enabling rapid kidney clearance and thereby improving biosafety—effectively addressing the limitations associated with the aforementioned materials.

### Phosphorus

Phosphorus, as an important benign element in the human body, accounts for approximately 1% of the total body weight and is the cornerstone of human bones [115]. Its functions and roles in living organisms cannot be ignored, especially in the field of tumor treatment, where it shows potential application value. Black phosphorus quantum dots (BPQDs) successfully synthesized by Lang et al. [116], as a novel nanomaterial, significantly enhanced the sensitivity of human RCC cells to radiation by inhibiting the activity of the DNA-dependent kinase catalytic subunit. This discovery provides a new strategy for RT of RCC. In vivo, the combined application of BPQDs and IR not only confirmed their potential for synergistic enhancement but also demonstrated significant efficacy superior to that of a single-treatment approach. This research not only reveals the potential application of phosphorus in tumor treatment but also opens up new approaches for the treatment of RCC.

Phosphorus plays a crucial role in the treatment of kidney injury. BPQDs and black phosphorus nanosheets, as phosphorus-based nanomaterials, possess unique chemical and physical properties that give them significant advantages in regulating oxidative stress, promoting cell repair, and enhancing biocompatibility. The NIR-II fluorescence imaging characteristics of BPQDs provide clinicians with a noninvasive real-time monitoring method, which is conducive to the early diagnosis and evaluation of treatment effects. BPQDs recently synthesized by Ge et al. [117] have shown significant potential in the field of renal injury treatment. These quantum dots can not only effectively resist ROS-mediated AKI but also have the ability to perform NIR-II fluorescence imaging, achieving real-time monitoring of kidney injury. Furthermore, Hou et al. [118] designed a black phosphorus nanosheet, which has kidney-targeting properties and can accumulate preferentially in the kidneys. This nanosheet significantly alleviated apoptosis by effectively eliminating ROS, providing a new strategy for the treatment of AKI (Fig. 3C). Black phosphorus nanosheets, through their kidney-targeting properties, can directly act on damaged renal tissues and effectively eliminate ROS, which is one of the main causes of renal cell damage and apoptosis. In addition, black phosphorus nanosheets can regulate intracellular signaling pathways, promote cell survival and repair, and thereby reduce the degree of kidney damage.

In contrast to selenium, phosphorus NPs (PNPs) exhibit intrinsically weak antioxidant capacity but function effectively as photosensitizers to induce PTT and PDT, rendering them more applicable to antitumor therapy than nephroprotection. Nevertheless, as an essential element for humans metabolizable through physiological pathways, PNPs demonstrate favorable biosafety profiles. These characteristics preclude their simultaneous application for both antitumor and kidney protective purposes. However, they can synergistically complement other elements within integrated therapeutic systems. In such architectures, distinct functional components assume specialized roles in either tumor eradication or kidney protection, operating without mutual interference or conferring modest auxiliary benefits, thereby generating synergistic effects to achieve genuine integrated therapeutics (Table 2).

**Table 2.** Inorganic nonmetallic biomaterials for RCC therapy and renal injury repair

Core material	Antitumor function			Nephroprotective function		
	Platform	Antitumor function	Key refs.	Platform	Nephroprotective function	Key refs.
Carbon	Reduced graphene oxide (rGO), multiwalled carbon nanotubes (MWCNTs)	rGO enhances RF-induced apoptosis; MWCNTs down-regulate HSPs to boost PTT efficacy	[84,88]	Graphene, carbon nanotubes	GO augments mesenchymal stem cells mediated recovery in cisplatin-AKI, carbon quantum dots preferentially accumulate in injured kidney	[92,93]
Selenium	Selenium nanoparticles	Induce ROS-mediated apoptosis in RCC cells and enhance therapy responsiveness	[100]	Selenium nanoparticles	Reduce oxidative stress, inflammation, and apoptosis, improve mitochondrial enzyme activity in AKI	[111]
Phosphorus	Black-phosphorus quantum dots	Radiosensitization by promoting DNA kinase activity	[116]	Black-phosphorus quantum dots	ROS scavenging, NIR-II imaging, renal targeting to alleviate AKI	[117]

RF, radiofrequency radiation; HSPs, heat shock proteins; PTT, photothermal therapy; ROS, reactive oxygen species; AKI, acute kidney injury; RCC, renal cell carcinoma; NIR, near-infrared

### The contradictions and balance strategies of inorganic nonmetallic materials in integrated therapy

Unlike metallic nanomaterials, which generally possess intrinsic antitumor properties, inorganic nonmetallic counterparts exhibit marked chemical stability and favorable biocompatibility, primarily functioning as drug delivery vehicles. Certain members of this category additionally demonstrate antioxidant capabilities or efficient photothermal conversion, enabling their integration into unified therapeutic systems. Through synergistic complementation of respective limitations, these hybrid architectures expand the strategic repertoire for integrated treatment modalities.

## Organic Materials for Integrated Therapy

Organic materials, with their significant advantages such as high stability, hydrophilicity, renewability, biodegradability, nontoxicity, safety, and low cost, as well as their rich active group characteristics, are widely used in drug delivery systems [119–121]. By enhancing the pharmacological activity of drugs, improving the chemical stability and water solubility of active molecules, optimizing biodistribution, controlling drug release rates, and increasing drug selectivity, organic materials can significantly improve the therapeutic effect of drugs and effectively reduce side effects (Table 3). Among them, controlling the drug release mechanism is the key to achieving cancer-specific drug delivery. By responding to various stimulating factors, such as pH value, light, temperature, enzyme activity, and ROS, organic materials can effectively reduce the premature release of drugs, lower the toxic effects on normal cells, greatly improve the side effects of drugs during the treatment, and enhance the accuracy and safety of treatment.

**Table 3.** Organic biomaterials for RCC therapy and renal injury repair

Core material	Antitumor function			Nephroprotective function		
	Platform	Antitumor function	Key refs.	Platform	Nephroprotective function	Key refs.
Lipid	Liposomes, lipid nanoparticles, solid–lipid nanoparticles, small extracellular vesicles	Drug delivery	[60,127,128,133]	Liposomes, lipid nanoparticles, extracellular vesicles, exosomes	Drug delivery, activates Keap1–Nrf2 pathway, immune modulation via exosome uptake	[134,139,141,143]
Chitosan	Chitosan	Drug delivery	[147]	Carboxymethyl chitosan, sulfated chitosan	Drug delivery	[156]
Hyaluronic acid	Hyaluronic acid hydrogel	Drug delivery	[159]	Hyaluronic acid-coated liposomes	Kidney targeting	[164]
Polylactic acid–glycolic acid	Polylactic acid–glycolic acid	Drug delivery	[169]	Polylactic acid–glycolic acid	Drug delivery	[174]
Polyethylene glycol	Polyethylene glycol–IL-2, polyethylene glycol–IL-10	Drug modification	[177,178]	Polyethylene glycol–gambogic acid	Drug delivery, targeted delivery	[183]

### Lipids

Lipid NPs (LNPs) are mainly classified into 5 categories, namely, liposomes, nano (micro)emulsions, solid lipid NPs (SLNs), nanostructured lipid carriers (NLCs), and exosomes [122–125]. Among them, liposomes are nanoscale vesicles derived from artificial phospholipid bilayer layers, featuring easy preparation, high biocompatibility, and highly tunable physical and chemical properties. They can undergo flexible functional modifications, such as targeting, stimulation response, and imaging, and can encapsulate almost all types of contents [126]. It is one of the most mature nanocarriers in the development of antitumor drugs.

Mo et al. [127] synthesized pH-sensitive oligopeptide liposomes for delivering tacrolimus. Compared with unpegylated liposomes, they have higher bioavailability and blood persistence and exhibit good antitumor efficacy. The lipid NPs of α-tocopheryl succinate (ssPalmE) synthesized by Akita et al. [128], after PEG modification, can enhance their stability in blood circulation, achieve tumor-selective gene expression, and inhibit the growth of RCC. Mao et al. [129] delivered lncRNA using liposomes as carriers, modified it with polydopamine (PDA) to enhance biocompatibility, improve targeting ability, and reduce toxicity, and simultaneously linked MUC12 antibodies to target RCC cells, which can effectively inhibit the growth of RCC metastasis in vivo. Liposomes, as delivery platforms, can also integrate multiple treatment methods, enhance the efficacy of tumor treatment, and improve the shortcomings of traditional treatment approaches. Abshire et al. [130] reported that thermosensitive liposomes loaded with TKIs can not only enhance the therapeutic effect on RCC through focused ultrasound (FUS) but also achieve controlled release of drugs, improve drug targeting, and have the functions of confocal and fluorescence imaging visualization of RCC uptake, assisting in the integrated diagnosis and treatment of RCC. The sertrabrin/indocyanine green (ICG)-loaded liposome Ser/ICG@Lip synthesized by Lei et al. [131] has excellent photoacoustic imaging and NIR fluorescence imaging capabilities and possesses both chemotherapy and PTT functions. Moreover, chemotherapy–photothermal combination therapy for RCC is more effective than either PTT or chemotherapy alone.

In the field of RCC treatment, various LNP technologies have been applied to drug delivery. For instance, researchers such as Sandhya et al. [132] developed a self-emulsifying drug delivery system for oral sorafenib. This system can significantly reduce first-pass metabolism and increase drug solubility, thereby enhancing the bioavailability of sorafenib by 5 times. On the other hand, the team led by Grillone utilized thermal homogenization technology to encapsulate sorafenib and superparamagnetic Fe_2_O_3_ NPs within SLNs, achieving a drug loading efficiency as high as 90% while maintaining good stability and biocompatibility [60]. In addition, exosomes/vesicles have also been used as a new strategy for the treatment of RCC. Özkan et al. [133] extracted small extracellular vesicles (SEVs) from garlic to induce apoptosis of RCC cells and reduce the expression of vascular endothelial growth factor (VEGF), a key protein for angiogenesis in cancer cells. These studies have provided new therapeutic ideas for overcoming the problem of drug resistance in RCC.

In renal injury, liposomes remain one of the commonly used drug delivery materials. Li et al. [134] used the pentapeptide CREKA, which can specifically bind to fibronectin, to modify liposomes to deliver celastrol. This can target renal fibroblasts, reduce their systemic toxicity and side effects, and enhance the antifibrotic effect. Zhou et al. [135] coated PLGA with a phospholipid bilayer to achieve passive targeting of the kidneys by adjusting the particle size and co-modified it with PEG and α8 integrin antibody to achieve active targeting for the delivery of dexamethasone (DXM) and captopril (CAP), which could significantly reduce their systemic toxicity and side effects and improve the anti-inflammatory and anti-fibrotic effects. Jung et al. [136] modified liposomes with epidermal growth factor antibodies for the delivery of cisplatin for RT sensitization, which could significantly alleviate cisplatin-induced nephrotoxicity. Chen et al. [137] delivered miR-155 inhibitors by liposomes, which could significantly enhance their colloidal stability and efficiently deliver them to renal tissues, effectively alleviating AKI. Microparticles (MPs) of kidney-derived MSCs (KMSCs) can improve the sparsity of capillaries around renal tubules by inhibiting endothelial–mesenchymal transition (EndoMT) and preventing the progression of renal injury by inhibiting tubulointerstitial fibrosis [138]. MSC extracellular vesicles (MSC-EVs) accumulate in renal tubules during renal ischemia/reperfusion (I/R) injury and promote renal function recovery by activating the Keap1–Nrf2 signaling pathway and enhancing the mitochondrial function of tubular epithelial cells (TECs) [139]. Cheng et al. [140] delivered the macrocyclic amphiphilic molecule C5A and the commercial dye sulfonated aluminum phthalocyanine (Pc) with MSC-EVs, which could enhance its therapeutic effect on AKI by inducing M1 to M2 macrophage transformation. Kim et al. [141] treated renal injury caused by I/R with a superrepressor inhibitor of NF-κB delivered by exosomes, which could reduce the activity of NF-κB in the kidneys after ischemia and decrease apoptosis. The uptake of exosomes by neutrophils and macrophages can also regulate the immune cell population of damaged kidneys, reduce the frequencies of neutrophils, monocytes/macrophages, and T cells, and inhibit inflammation. Wu et al. [142] designed peptide-coupled tripterygiol (CLT)–phospholipid lipid nanoparticles (PC-PLNs), which can efficiently deliver CLT to damaged endothelial cells and podocytes, effectively alleviating CKD. Hou et al. [143] delivered SOD_2_ mRNA using LNPs to alleviate AKI by regulating mitochondrial ROS levels.

Compared to other organic nanomaterials, liposomes possess the distinct advantage of superior clinical translatability. The success of COVID-19 mRNA vaccines signifies the maturity of liposomal formulations in commercial manufacturing workflows, from scalable production to regulatory approval. Employing liposomes as delivery vehicles for integrated therapeutic systems would substantially accelerate their clinical translation efficiency.

### Chitosan

Chitosan (CS) has biocompatibility, biodegradability, and a simple and mild synthesis process and thus is widely chosen in the pharmaceutical industry as a compound for delivering therapeutic drugs [144,145]. Moreover, CS oligosaccharide can induce G2/M phase arrest and apoptosis in a ROS-dependent manner and activate the ERS signaling pathway. It has an inhibitory effect on the proliferation of human RCC both in vitro and in vivo [146]. Therefore, Kavi Rajan et al. [147] used CS as a vector and loaded chlorogenic acid (CGA) to treat RCC, significantly enhancing the intracellular accumulation, antiproliferative activity, and antioxidant properties of CGA.

As a biomaterial with excellent anti-inflammatory properties and self-assembly ability, CS has shown significant application potential in the field of renal injury treatment [148]. Studies have shown that low-molecular-weight CS (L-CS) NPs, due to their cationic surface characteristics, can effectively prolong the in vivo circulation time. Furthermore, considering that the glomerular basement membrane (GBM) carries a relatively high negative charge, L-CS NPs have demonstrated specific targeting for the kidneys [149]. Aydin et al. [150] achieved passive targeting to the kidneys by regulating the size of CS NPs, thereby significantly enhancing the delivery efficiency of small interfering RNA (siRNA). At the molecular level, Yang et al. [151] used CS as a carrier to deliver siRNA targeting COX-2 to macrophages, effectively reducing the levels of oxidative stress, inflammatory response, and apoptosis, thereby alleviating the progression of renal fibrosis. CS itself also has the ability to promote tissue repair. Crab CS NPs prepared by researchers, such as Shati et al. [152], can significantly improve kidney injury and restore it to a state close to that of normal cells. In terms of drug delivery, the application of CS has significantly improved the pharmacokinetic properties of drugs and enhanced their stability in vivo. Zhang et al. [153] also demonstrated that methoxy PEG CS NPs can enhance the oral stability of salvianolic acid B and increase its mucus penetration ability and membrane transport efficiency in the gastrointestinal tract, thereby improving its bioavailability and alleviating renal fibrosis in mice. Further research has expanded the application fields of CS. For instance, Wang et al. [154] constructed NPs using triphenylphosphine and L-CS and, through internalization mediated by Megalin receptors, achieved specific targeting of renal TECs, effectively reducing the generation of oxidative stress and thereby treating AKI. Liu et al. [155] enhanced the ability of KIM-1 to target damaged kidneys by modifying CS with L-serine and further strengthened the therapeutic effect of SS31 by taking advantage of the ROS responsiveness of thioketal. Tang et al. [156] demonstrated the application of CS in the delivery of p53-siRNA. By modifying CS with α-cyclam-p-toluic acid (C-CS), targeting was enhanced, and the CXCR4-mediated immune response was inhibited, significantly increasing the accumulation of p53-siRNA in the kidneys and the therapeutic effect. These studies collectively demonstrate that CS has broad application prospects in the treatment of renal injury, providing strong support for the development of new therapeutic strategies.

The amino groups on the CS surface become positively charged at physiological pH, while abundant functional moieties enable diverse chemical modifications for tailored functionalities. Notably, L-CS undergoes renal clearance without accumulation toxicity and exhibits intrinsic renal targeting capability, making it the preferred choice for integrated therapeutic applications.

### Hyaluronic acid

Hyaluronic acid (HA), as a glycosaminoglycan in the extracellular matrix (ECM), plays a key role in fundamental cellular and molecular biological processes such as cell signal transduction, immune regulation, tumor progression, and angiogenesis [157]. The signal transduction and functional properties of HA depend on its molecular weight. Among them, high-molecular-weight HA (HMW-HA; >500 kDa) promotes anti-inflammatory effects, while low-molecular-weight HA (LMW-HA; <120 kDa) serves as a key component in triggering local inflammatory signals [158]. Ueda et al. [159] innovatively designed a HA-tyramine (HA-Tyr) hydrogel to carry interferon-α (IFN-α). The research results revealed that this hydrogel could significantly extend the biological half-life of natural human IFN-α and enhance its antitumor effect on human RCC cells. This discovery highlights the potential of HA in innovative tumor treatment strategies, especially by extending the bioavailability of drugs and enhancing their biological activity, thereby playing a key role in the treatment of RCC.

HMW-HA can exert cell-protective and antifibrotic effects, reducing the formation of tubulointerstitial scars in renal injury [160]. Qin et al. [161] prepared hydrogels using HA derivatives for long-acting controlled-release anti-inflammatory small-molecule celastrol or anti-transforming growth factor-β (TGF-β) antibodies. These gels can inhibit the NF-κB signaling pathway or locally neutralize TGF-β1 to alleviate fibrosis, enhancing the therapeutic effect of the drug without any local or systemic toxicity. Hu et al. [162] modified HA with CD44 to load curcumin, which could increase the renal accumulation of curcumin by 13.9 times through targeted action, enhance its antioxidant capacity to inhibit the PtdIns3K–AKT–mTOR pathway, and alleviate AKI. Huang et al. [163] reported that NPs (nHA/PLBR) coated with HA for delivering ε-polylysine–bilirubin conjugates (PLBR) could significantly enhance the targeting of PLBR, promote its accumulation in damaged kidneys, and improve the therapeutic effect of AKI. Huang et al. [164] developed a HA-coated liposome targeting renal epithelial cells for the delivery of curcumin, which can be targeted and released according to the acidic microenvironment of the lesion to enhance the targeting ability and bioavailability of curcumin. Zhou et al. [165] conjugated HA with 4-methoxyphenylthiouurea (MPT) and thione bonds (2′-[propan-2,2-diyllBLs (thiodide)] diacetic acid, TKL) to construct HATM NPs and modified them with CD44 antibody, in which MPT can produce H_2_S in vivo to alleviate AKI. TKL has ROS responsiveness and, together with CD44, can further enhance the vector targeting ability. The loading of rapamycin (RAP) with HATM can significantly enhance its biocompatibility and cellular permeability, treating renal I/R injury.

CD44 serves as the primary receptor for HA, exhibiting high expression in cancer stem cells (CSCs), tumor cells undergoing epithelial–mesenchymal transition (EMT), and inflamed/fibrotic kidneys, while remaining low in normal tissues. This differential expression endows HA with intrinsic active targeting capabilities toward both oncological and renal injury lesions. Furthermore, the self-assembly properties of HA provide versatile design strategies for integrated therapeutic systems. Additionally, HA can function as an in situ injectable hydrogel carrier, rendering it particularly suitable for localized drug delivery scenarios such as postoperative sites or percutaneous puncture interventions.

### Polylactic acid–glycolic acid

Polylactic acid–glycolic acid (PLGA) has been approved by the FDA as a drug carrier due to its hydrophobicity and biocompatibility. Ordikhani et al. [166] modified PEGylated PLGA NPs with λ light chains (LCs). Through their interaction with the membrane protein meggalin, they selectively target proximal TECs (PTECs) of the kidney and specifically retain them in PTECs for 7 d. Kim et al. [167] encapsulated Toll-like receptor 7/8 (TLR7/8) agonists with PLGA, which increased the costimulatory molecule expression and antigen presentation of dendritic cells through major histocompatibility complex class I (MHC I). When administered subcutaneously in mice, it could produce significant preventive and therapeutic effects in the RCC model. Lee et al. [168] used PLGA loaded with cabozantinib. In vivo experiments showed that it reduced the lung metastasis burden of mice and prolonged their lifespan, demonstrating good safety. Hamedani et al. [169] equipped PLGA with Honokiol and coupled it with electrospun polymeric fibrous scaffolds. This complex has a good sustained-release effect and can inhibit the proliferation and migration of RCC cells in vitro.

As a multifunctional polymer material, PLGA has demonstrated unique advantages in the treatment of renal injury. It can effectively address issues such as poor bioavailability and poor in vivo stability of drugs, significantly enhancing the therapeutic effects of drugs on renal injury and fibrosis [170]. Further modification of PLGA can endow it with diverse functions and further enhance the therapeutic effect. Sun et al. [171] modified PLGA NPs with PEG and cell-penetrating peptides (CPPs) to enhance their bioavailability and targeting ability and used them to deliver tanshinone IIA (Tan IIA) to construct Tan IIA NPs. Tan IIA NPs and curcumin were encapsulated with CS-alginate microcapsules to protect CPPs from degradation in the gastrointestinal tract and prevent curcumin from being released prematurely under acidic conditions. This system can significantly improve the targeting ability and bioavailability of oral Tan IIA and curcumin and enhance their therapeutic effect on AKI. Yao et al. [172] delivered ICG and SS31 using PLGA and wrapped them with platelet membranes. ICG can be used for the NIR diagnosis of AKI, SS31 can be used for the treatment of renal injury, and the platelet membrane enables the system to target the kidneys, increase their bioavailability, and achieve the integration of diagnosis and treatment of AKI. Wei et al. [173] loaded peroxisome proliferator-activated receptor γ (PPARγ) agonists with PLGA and combined them with SonoVue microbubbles (MBs). Under ultrasound (US) exposure, MBs can enhance the permeability of local cell membranes or microvascular systems, improve the targeting effect of NPs, and thereby enhance the antifibrotic effect of PPARγ agonists. Alotaibi et al. [174] delivered epigallocatechin-3-gallate (EGCG) with PLGA to address the issues of poor intestinal stability and permeability, as well as low oral utilization, and to enhance its renal protective ability. Hu et al. [175] designed DXM-loaded e-selectin targeting sialic acid PEG DXM (SA-PEG-DXM/DXM)-conjugated micelles for the treatment of AKI, which can extend the DXM release time to 48 h. E-selectin can target SA expressed on endothelial cells, allowing it to accumulate in damaged kidneys. SA in the conjugate can reduce toxicity by inhibiting lipopolysaccharide (LPS)-activated Beclin-1/ATG5-ATG12-mediated autophagy, thereby significantly improving LPS-induced proinflammatory cytokine production.

PLGA, as a relatively mature biocompatible material for clinical application, is a classic biodegradable polyester material approved by the FDA. It can be used as a carrier to improve the pharmacokinetics of drugs and enhance therapeutic efficacy.

### Polyethylene glycol

As early as 20 years ago, clinical trials explored PEG-modified IL-2 for the treatment of RCC. The results showed that PEG had good biocompatibility, but its antitumor activity did not increase significantly [176]. Motzer et al. [177] also conjugated PEG with IL-2α, which could reduce drug toxicity and improve efficacy by altering the pharmacokinetic characteristics of the drug but ultimately did not achieve significant efficacy enhancement. Therefore, researchers continued to explore the therapeutic effect of PEG-modified IL-10 on RCC. The preliminary results indicated that PEG–IL-10 had acceptable toxic characteristics and antitumor activity. There will be subsequent explorations of combined immunotherapy with PEG–IL-2 and PEG–IL-10. Combined immunotherapy with PEG–IL-10 has shown promising clinical activity [178], and the results also indicate that PEG has negligible toxicity in RCC, laying a good foundation for the subsequent application of PEG in RCC treatment [179].

PEG plays a crucial role in the treatment of renal injury. First, PEG can modify drug molecules to form NPs, improving the solubility and stability of drugs and increasing the circulation time of drugs in the body. Imig et al. [180] coupled stable epoxy-eicostrienoic acid (EET) analogs to folic acid through PEG-diamine linkers, which could significantly enhance the therapeutic effect of EET, achieving double the effect with only ^1^/_10_ of the dose. Xu et al. [181] fabricated NPs by binding the C domain of insulin-like growth factor 1 (IGF-1C) with 1,2-distearoyl-Sn-glycerol-3-phosphoethanolamine-N-[maleimide (polyethylene ethyl acetyl)] (DSPE-PEG-MAL), which could enhance the antioxidant, anti-inflammatory, and anti-apoptotic capability properties of IGF-1C. Tong et al. [182] prepared NPs to deliver insulin using PEG-B-poly [(2-aminoethyl-l-glutamic acid)-G-poly (l-lysine)] [PEG-b-P (ELG-g-PLL)], which could improve the sustained release and higher bioavailability and enhance its protective effect. Second, PEG helps enhance the targeting of drugs, enabling them to act more precisely on the site of kidney injury and improve the therapeutic effect. In addition, PEG can also promote intracellular endocytosis of drugs, enhance the effects of drugs within cells, and further improve the effectiveness of treatment. Li et al. [183] modified gambogic acid (GA) with PEG and self-assembled it into NPs of approximately 4.5 nm, which greatly enhanced the targeting and bioavailability of GA. Qin et al. [184] loaded CLT with D-α-tocopherol PEG 1000 succinate (TPGS) and modified it with bovine serum albumin, which could enhance the targeting ability of CLT and promote endocytosis, thereby enhancing the therapeutic effect.

PEG is also a common organic material that mainly serves an auxiliary function. It is used to modify other materials, extend their half-life, reduce protein binding in plasma, etc., and has excellent biological safety. In the design of the integrated treatment, the addition of PEG can be considered to optimize the entire system.

### The contradictions and balance strategies of organic materials in integrated therapy

Owing to their intrinsic biocompatibility and low immunogenicity, and being largely devoid of inherent therapeutic activity, organic materials predominantly function as drug delivery vehicles. Consequently, they are rarely entangled in the paradox between therapeutic potency and kidney toxicity. Although not serving as the core therapeutic modality, their programmable surface chemistry, established clinical translation pathways, and versatility in formulation render them indispensable components in the rational design of unified integrated systems. It is also the key to designing the response structure of the microenvironment.

## Challenges and Design Strategies for Integrated Therapy of RCC and Kidney Injury

The integration of antitumor therapy and nephroprotection into a unified therapeutic paradigm represents a critical unmet need in oncological and nephrological medicine, particularly for patients with preexisting CKD or chemotherapy-induced renal injury. However, this dual-objective approach faces inherent contradictions at the materials science and biological levels. First and foremost is the therapeutic modality paradox: Effective tumor eradication typically requires cytotoxic agents, prolonged tissue retention, or metal-based nanomaterials (e.g., Au, Fe, Cu, and Se) with intrinsic photothermal, chemodynamic, or nutritional lethal activities, whereas renal protection mandates antioxidant, anti-inflammatory, or regenerative interventions that must be rapidly cleared or metabolically inert to avoid exacerbating renal burden. This fundamental conflict is compounded by the biodistribution dilemma—antitumor efficacy relies on the enhanced permeability and retention (EPR) effect and reticuloendothelial system evasion for accumulation in tumor sites, yet such long-circulating, nondegradable materials (e.g., CNTs and AuNPs) inevitably accumulate in the kidneys or liver, potentially inducing nephrotoxicity or fibrosis. Furthermore, the metabolic incompatibility between cytotoxic metal ions (e.g., Fenton-active Fe/Cu) and the delicate redox balance required for renal TEC survival creates a narrow therapeutic window where pro-oxidant tumoricidal mechanisms may exacerbate IR or drug-induced kidney injury.

To circumvent these obstacles, rational design strategies must transcend simple material selection toward spatiotemporally decoupled architectures. One promising approach involves environment-responsive structural transformation. These design strategies can be classified into endogenous and exogenous responsiveness. For endogenous responsiveness, one approach involves engineering TME-responsive polymer conjugates that release cytotoxic payloads under acidic conditions characteristic of tumors while retaining antioxidant properties at the neutral pH of healthy renal tissue. The CeO-NPs designed by Weng et al. [185] can regulate ROS and restore redox homeostasis by decomposing hydrogen peroxide and activating the Nrf2/Keap1 signaling pathway to protect renal tubules. However, in the acidic tumor microenvironment within the tumor, it becomes inert, allowing chemotherapy-mediated ROS generation and accumulation to kill cancer cells. It has environment-dependent catalytic activity and holds great potential in the clinical prevention and treatment of AKI in cancer patients (Fig. 3D). The pH-responsive BP@CeO_2_-PEG designed by Gao et al. [82] exhibits activity similar to that of multiple antioxidant enzymes in a physiologically neutral environment, effectively eliminating ROS and protecting the kidneys from oxidative stress damage. However, in the acidic microenvironment of tumors, H^+^ ions in the complex can inhibit the transformation of Ce^4+^ to Ce^3+^, disrupt the redox cycle, lead to the loss of ROS clearance ability, cause ROS accumulation within the tumor, and kill tumor cells. Exogenous responsiveness, by contrast, harnesses external stimuli such as light, ultrasound, or heat to activate therapeutic modalities including PDT, PTT, and SDT. Under these stimulated conditions, antitumor effects are induced specifically at the lesion site, whereas in unstimulated normal kidney tissues, the materials maintain their inherent antioxidant characteristics, effectively achieving spatial segregation of cytotoxic and protective functions. For example, Kroeze et al. [186] indicated that the photosensitizer meso-tetra(hydroxyphenyl)chlorin can protect normal renal tissue outside the illuminated area, providing a new option for protecting normal nephrons in the treatment of small renal tumors.

Hybrid systems incorporating multiple low-toxicity materials offer an alternative paradigm. In such configurations, inorganic cores furnish tumor-specific photothermal or chemotherapeutic functionalities while concurrently housing nephroprotective agents, with the diverse components operating synergistically yet without mutual interference. Finally, an organic shell (such as liposomes, PLGA, and CS) is encapsulated on top. These shells give the entire system excellent biocompatibility, tumor targeting, stealth properties, and controllable clearance capabilities. This architectural compartmentalization ultimately enables each constituent to perform its specialized function, accomplishing the integrated objectives of antitumor efficacy and kidney protection. Jiang et al. [187] combined rGO with gallic acid to treat RCC through RF, demonstrating a good antitumor effect while causing no damage to normal cells. The research by Huang et al. [188] demonstrated that peptide amphiphile micelle vectors have excellent renal targeting properties, can accumulate in the kidneys without damaging tissues, and have been used in gene therapy for RCC, effectively inhibiting tumor growth and metastasis.

Finally, therapeutic sensitization enables dose reduction of existing treatments, thereby mitigating renal injury. For instance, in chemotherapy, human organic cation transporter 2 (OCT2) is the most abundant and important uptake transporter involved in the renal excretion of cationic drugs. The inhibition of OCT2 expression is a key factor leading to decitabine (DAC) resistance [189]. Chen et al. [190] indicated that the hypoxic environment within tumors can lead to the up-regulation of histone deacetylase HDAC9, which impairs the enrichment of H27K2ac modification in the OCT2 promoter and subsequently causes transcriptional inhibition of OCT2. Hypoxia-mediated human ENT1 inhibition is significantly inhibited in RCC, leading to a reduction in DAC cell accumulation. Therefore, Chen et al. synthesized an oxygen nanocarrier based on hemoglobin, which can weaken the hypoxia-induced loss of DAC activity and make RCC cells sensitive to sequential combination therapy of DAC and oxaliplatin, based on the characteristic of DAC resistance caused by hypoxia in RCC. Wang et al. [191] developed an fibronectin-targeted self-assembled peptide ECM deprivation system that can target and recognize FN to form nanofibers, achieving long-term retention in the ECM and enhancing chemotherapy sensitivity by improving drug resistance mediated by cell adhesion. In the immunotherapy, the liposome tumor vaccine developed by Roth et al. [192] can induce long-lasting and effective systemic immunity by delivering 2 HER2 epitope peptides, enabling mice to resist subcutaneous and tail vein tumor loads. Moreover, when used to treat RCC, it also has a significant inhibitory effect on RCC. The lipid NPs constructed by Jing et al. [193] can efficiently introduce double circular RNAs into macrophages, forming chimeric antigen receptor macrophages expressing in situ engineered chimeric IL-2 signaling receptors. In the targeted therapy, the cureous oxide NPs synthesized by Yang et al. [68] can not only kill tumor cells but also down-regulate the expression of AXL, MET, AKT, and extracellular signal–regulated kinase (ERK) to restore sunitinib reactivity in sunitinib-resistant (SR) RCC cells. It may help develop promising new approaches to treat patients with acquired SRRCC.

## The Safety of the Material and Its Clinical Translational Capability

The kidney serves as one of the primary organs for biomaterial metabolism, and renal pathologies inherently alter their biodistribution. In AKI or CKD, reduced GFR prolongs material retention and increases tubular exposure at given systemic doses, thereby elevating toxicity risks. Consequently, the intrinsic biosafety and pharmacokinetic profiles of materials must be carefully considered. Overcoming the translational gap requires material designs that are not only efficacious but also clinically feasible and scalable. Key considerations include:

1. Administration route: The choice between systemic (intravenous) and localized (e.g., intratumoral injection and implantable hydrogels post-nephrectomy) delivery is critical. Systemic administration relies on passive (EPR) or active targeting, which faces challenges of heterogeneity and off-target accumulation. Localized delivery maximizes tumor exposure while minimizing systemic renal exposure, offering a direct strategy to decouple the spatial conflict between therapy and protection, particularly for localized RCC or adjuvant settings.
2. Learning from approved nanomedicines: The clinical journey of liposomal doxorubicin (Doxil) and Fe_2_O_3_NPs (ferumoxytol) provides invaluable lessons. Doxil’s reduced cardiotoxicity but altered (sometimes increased) renal handling underscores the need for organ-specific toxicity reevaluation. Ferumoxytol, an FDA-approved USPIO, demonstrates the safety of systematic Fe_2_O_3_ use and its potential as a magnetic resonance imaging contrast agent and drug carrier prototype for renal applications.
3. Manufacturing and control: Translation demands robust, reproducible synthesis (e.g., using microfluidics for lipid NPs) and strict control over critical quality attributes (CQAs) like size, charge, drug loading, and release kinetics—parameters that directly influence both efficacy and nephrotoxicity. Furthermore, clinical translation entails additional hurdles including scalable manufacturing, batch-to-batch reproducibility, economic viability, and navigating regulatory approval pathways. These factors place substantial demands on material selection and fabrication processes. Employing materials with proven clinical translation histories (for example, liposomes) can mitigate translational barriers and accelerate the path to clinical implementation.
4. Safety-first pharmacology: Preclinical studies must move beyond proof-of-concept efficacy to include Good Laboratory Practice (GLP)-compliant toxicology studies, detailed biodistribution analyses in models with preexisting renal impairment, and assessment of immune responses (e.g., complement activation-related pseudoallergy).

Overall, by constructing sustained-release carriers such as hydrogels for local delivery in postoperative or percutaneous puncture clinical scenarios, overall biosafety can be significantly enhanced. For example, PEG not only optimizes the pharmacokinetic properties of drugs, reduces toxicity, and enhances efficacy but also has been used to treat RCC in multiple clinical trials. In addition, PEG has also been applied in perioperative kidney protection studies. For instance, research by Staehler et al. [194] demonstrated that the use of collagen patches coated with PEG significantly reduced blood loss and operation time during partial nephrectomy without affecting renal function. Concurrently, comprehensive safety assessments—including monitoring hematological parameters, serum creatinine, and blood urea nitrogen—are essential to ensure material safety, representing a critical prerequisite for clinical translation. Finally, utilizing materials with established clinical translation track records will substantially reduce translational barriers and improve clinical implementation efficiency.

## Conclusion

**Fig. 4.**
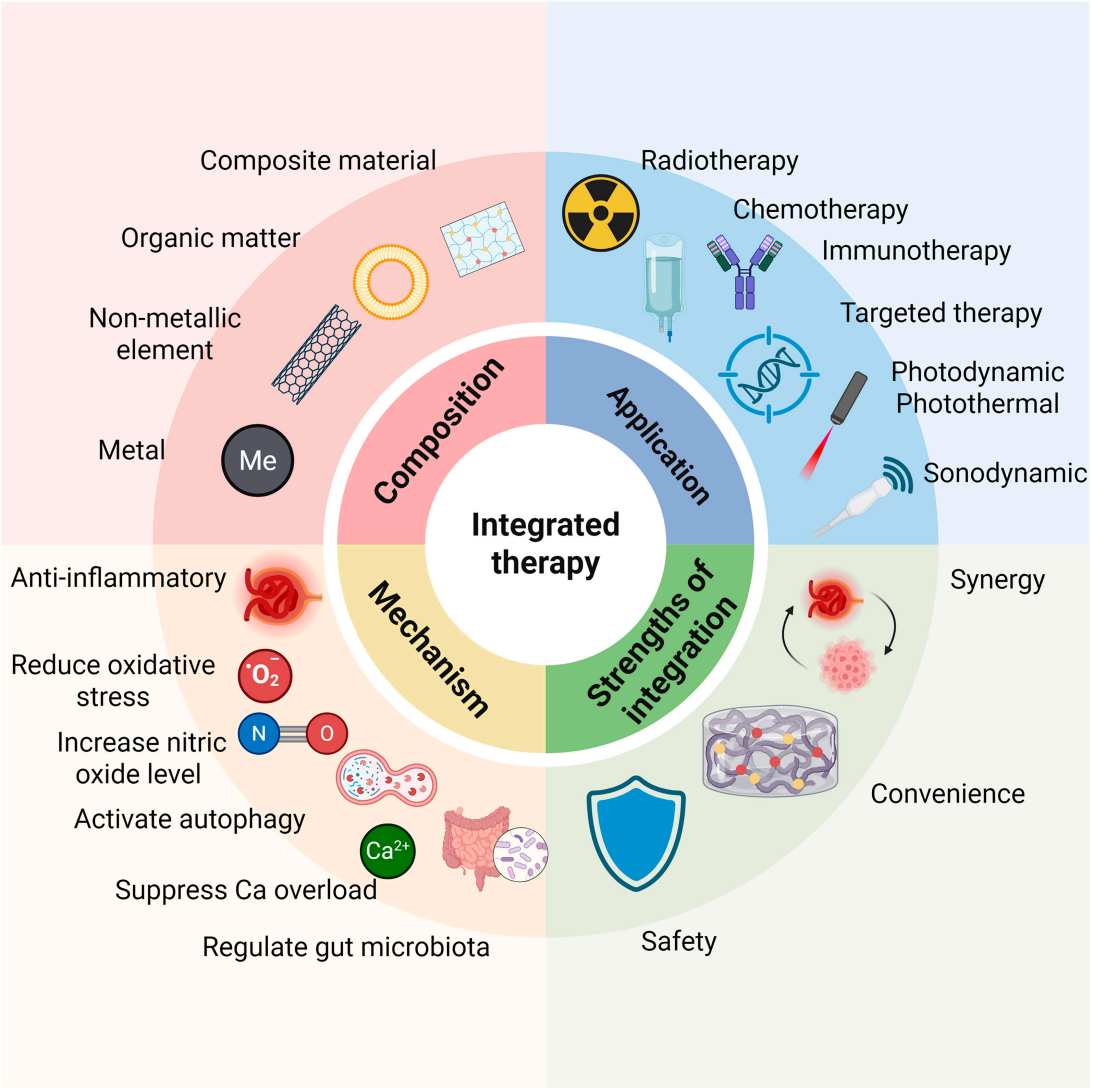
Schematic diagram of the integrated strategy for antitumor and kidney protection. Drawing on a rich biomaterial repertoire, material combinations are assembled through multiple therapeutic modes and mechanisms to realize an integrated strategy for antitumor and kidney protection. This unified approach organically links tumor suppression with functional preservation, offering greater synergism, convenience, and safety (created with BioRender).

In the current field of medical research, balancing cancer treatment and kidney protection has become an important trend in future clinical treatment. This article innovatively proposes the material design concept of “antitumor and kidney protection”, aiming to achieve a synergistic effect between cancer treatment and kidney repair by integrating different types of materials, thereby significantly improving the long-term prognosis of patients (Fig. 4). We delved deeply into the material design concepts and mechanisms of action for the treatment of RCC and renal repair from 4 dimensions: metallic materials, inorganic nonmetallic materials, organic materials, and composite materials. While exploring the design of “antitumor and kidney-protecting” materials, we have recognized the limitations of the current research. At present, research on the treatment of RCC and renal injury often focuses on a single goal and rarely considers the mutual influence between the 2 simultaneously. The research is still in its infancy and faces many challenges.

First, biocompatibility is an indispensable cornerstone in material design. In the integrated treatment of antitumor and kidney protection, multiple materials need to work in synergy, being both effective for tumor cells and safe for normal cells. This requires that, while improving the therapeutic effect, the balance between efficacy and toxicity be ensured. The realization of this goal undoubtedly increases the complexity of material screening and preparation.

Second, the compatibility of material functions is also an important consideration. Although some materials have shown effects in both cancer treatment and kidney damage repair, their mechanisms may be completely different. For instance, in tumors, ROS are produced to kill tumor cells, while in kidney tissue, ROS need to be eliminated to protect the kidneys. This may cause conflicts in the design of material functions, and we need to further enhance our research on targeted delivery and controlled-release technologies of drug carriers to ensure that materials perform their correct functions in the right positions.

Finally, the scarcity of ligands targeting specific renal cell types and RCC limits the targeting of drugs. This requires us to delve deeply into efficient molecular markers of kidney damage and RCC to enhance the targeting of drugs and thereby achieve more precise treatment.

**Fig. 5.**
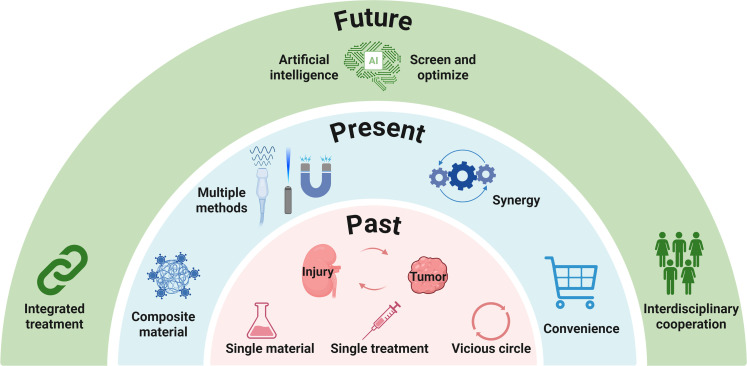
The past, present, and future of integrated antitumor and kidney protection strategies. Biomaterials in RCC have evolved from single-material therapies in the past to current multimaterial combinations that integrate diverse treatment modalities, yet preservation of organ function remains largely overlooked. Future advances will incorporate AI-assisted biomaterials and databases to develop integrated antitumor and kidney protection material combinations. These strategies will not only eradicate renal tumors but also promote renal repair, disrupting the vicious cycle between renal injury and RCC. This holistic approach promises safer, more effective and comprehensive therapy, offering new directions for RCC treatment (created with BioRender).

As shown in Fig. 5, the future transformation and application of “antitumor and kidney-protecting” integrated materials in clinical practice will require interdisciplinary collaboration. The rapid development of artificial intelligence technology will bring new opportunities for integrated treatment [195]. By combining biological material and drug information databases with artificial intelligence-assisted design and high-throughput screening, especially in the aspect of responsive linker design, pharmacokinetic prediction, and toxicity screening, integrated treatment plans can be quickly discovered and optimized, greatly improving the efficiency of material design. At the same time, the joint efforts of multiple forces, including materials scientists, clinicians, patients, basic scientists, and government agencies, are also needed. Through comprehensive and in-depth research, it is expected that a synergistic effect of treatment and protection can be achieved in the future, providing patients with more humanized and effective treatment plans and promoting the innovation and progress of medical technology.
